# York-Mason Repair of a Rectourethral Fistula in a Hostile Abdomen: A Case Report

**DOI:** 10.7759/cureus.114311

**Published:** 2026-08-10

**Authors:** Luis Eduardo Morillo Lemus, Cesar Milton Gerardo Benito

**Affiliations:** 1 Department of Surgery, Clinica Abasolo, Moroleón, MEX; 2 Department of Gastrointestinal Surgery, Servicios de Salud del Instituto Mexicano del Seguro Social para el Bienestar, Poza Rica, MEX

**Keywords:** hostile abdomen, prostatectomy complication, rectourethral fistula, transsphincteric repair, york-mason procedure

## Abstract

Rectourethral fistula is an uncommon but complex complication that may occur after prostate surgery and often requires individualized surgical management. The York-Mason transsphincteric approach represents an option for selected patients, particularly when previous abdominal procedures make conventional approaches challenging.

This article describes the treatment of a rectourethral fistula using the York-Mason transsphincteric repair technique. The different surgical stages of this technique will be detailed, as an alternative to the management of rectus vesical fistula with hostile abdomen due to multiple surgical events.

We present a case of rectourethral fistula as a complication of radical prostatectomy in a patient with multiple previous abdominal surgeries, for which a transrectal-transsphincteric approach with the York-Mason technique was used, with satisfactory evolution and without complications during the postoperative period and with short-term follow-up.

A difficult case is shown in the management of rectal-urinary fistula with multiple abdominal surgeries, for which an alternative technique to the abdominal approach is performed with satisfactory results maintaining fecal continence and without postoperative complications.

For patients with multiple surgical events where lysis of adhesions by sharp/blunt dissection could cause preventable organic damage, an alternative access to the conventional one is necessary to perform fistula repair. This technique has the advantage of offering rapid exposure with a large working space and low morbidity. Careful repair of the anal sphincter muscle groups in this technique will allow complete continence.

## Introduction

Rectourethral fistulas are uncommon but complex complications that frequently require individualized surgical management. The York-Mason transsphincteric approach represents an alternative technique, particularly in patients with hostile abdomens due to previous abdominal procedures. We present the case of a 52-year-old male patient with a rectourethral fistula following radical prostatectomy and multiple subsequent abdominal interventions. Due to extensive intra-abdominal adhesions and previous failed repair attempts, a posterior transsphincteric York-Mason approach was performed. The fistulous tract was resected and repaired in three layers, followed by the anatomical reconstruction of the anal sphincter complex. The patient had an uneventful postoperative course, preserved fecal continence, and complete resolution of urinary contamination symptoms during follow-up. The York-Mason technique remains a valuable option in selected patients, especially when conventional abdominal approaches present increased risks due to hostile abdominal conditions.

Rectourethral fistula is a rare but severe complication after prostate surgery. Historically, reported rates after open retropubic or perineal prostatectomy ranged from 1% to 11%; however, with the widespread adoption of robotic-assisted techniques, the incidence has decreased substantially, with reported rates of approximately 0.17-0.53%. Despite this lower incidence, rectourethral fistula may still occur after robotic procedures due to factors such as unrecognized rectal injury, tissue ischemia, infection, delayed diagnosis, or impaired healing. In the present case, the development of this complication after robotic-assisted prostate surgery highlights that although minimally invasive approaches reduce risk, they do not completely eliminate the possibility of fistula formation.

Although the York-Mason technique is an established approach for rectourethral fistula repair, its role in patients with a hostile abdomen and multiple previous unsuccessful interventions remains clinically relevant. This report highlights the application of this approach in a complex patient with extensive prior abdominal surgery, recurrent fistula management challenges, and preservation of functional outcomes after definitive repair.

## Case presentation

The definitive diagnosis was a rectourethral fistula, defined as an abnormal communication between the rectum and the urethra. The term rectovesical fistula was not applicable in this case because the urinary communication involved the urethra rather than the urinary bladder.

The patient was a 52-year-old man with a history of long-standing arterial hypertension, controlled with telmisartan 40 mg daily. He had no history of diabetes mellitus, smoking, or pelvic radiotherapy. His relevant surgical history began with robotic-assisted prostate surgery (Da Vinci system) for benign prostatic hyperplasia in September 2017. The postoperative course was complicated by the development of a rectourethral fistula, followed by multiple abdominal interventions, including attempted fistula repair, diverting colostomy, and damage-control surgery, resulting in extensive intra-abdominal adhesions.

Seven days after the procedure, the patient developed a rectourethral fistula, clinically suspected due to the presence of enteric content in the urinary drainage bag. Subsequent clinical evolution was characterized by fecaluria, pneumaturia, recurrent urinary tract infections, and persistent urinary leakage through the rectum. 

The diagnosis of rectourethral fistula was established based on the postoperative clinical presentation, including the presence of enteric content in the urinary drainage system, followed by persistent fecaluria, pneumaturia, recurrent urinary tract infections, and urinary leakage through the rectum. The available clinical records did not provide detailed endoscopic characterization of the fistulous tract or documentation of vesicourethral anastomotic involvement.

The decision to perform a posterior transsphincteric York-Mason repair was based on the diagnosis of rectourethral fistula, the history of multiple previous abdominal interventions, and the presence of extensive intra-abdominal adhesions that increased the complexity and potential risk of another abdominal approach.

The fistulous tract was resected and repaired in three layers, followed by the anatomical reconstruction of the anal sphincter complex. The patient had an uneventful postoperative course, with complete resolution of urinary contamination symptoms, preserved fecal continence, and satisfactory functional recovery during follow-up. 

The patient's clinical course is summarized in Table 1.

**Table 1:** Chronological timeline of major clinical events in a patient with rectourethral fistula treated with the York-Mason technique The table summarizes the chronological sequence of major clinical events, including the initial prostatectomy, diagnosis of rectourethral fistula, previous surgical interventions, complications, definitive York-Mason repair, and postoperative follow-up.

Date/period	Clinical event
September 2017	Robot-assisted radical prostatectomy (Da Vinci system) was performed for benign prostatic hyperplasia.
7 days after prostatectomy	The patient developed a rectourethral fistula, evidenced by enteric content in the urinary drainage bag.
Shortly after diagnosis	Initial laparoscopic attempt at fistula repair was performed, including diverting colostomy.
7 days after the initial repair	The patient was readmitted with acute abdomen due to colorectal stump anastomotic dehiscence and intra-abdominal collection.
Subsequent hospitalization	Damage-control surgery was performed, including Bogotá bag placement and planned reoperation (second-look laparotomy) at 48 hours, followed by definitive abdominal wall closure.
After multiple unsuccessful conservative treatments	Persistent fecaluria, pneumaturia, recurrent urinary tract infections, and urinary leakage through the rectum led to definitive surgical management.
Definitive treatment	Posterior transsphincteric York-Mason repair of the rectourethral fistula was performed with three-layer closure and anal sphincter reconstruction.
Postoperative follow-up	Contrast enema demonstrated complete rectal integrity without leakage.
Six months after York-Mason repair	Colostomy reversal was performed.
Current follow-up	The patient has normal spontaneous urination and complete fecal continence.

Surgical technique

Before definitive repair, urinary diversion was achieved with urethral catheterization. Suprapubic catheterization was not performed. This approach was selected according to the treating team's assessment and the clinical evolution of the patient before definitive repair.

The patient underwent a posterior transsphincteric York-Mason approach under general anesthesia. A 20 Fr Foley catheter was placed, and the patient was positioned in the prone jackknife position. A posterior midline incision was performed, followed by division of the anal sphincter complex and dissection through the intersphincteric plane. Coccygectomy was performed to improve surgical exposure due to the complex anatomy and extensive previous surgical history. The fistulous tract between the rectum and urethra was identified and excised.

The repair was performed in a multilayer fashion. The urethral defect was closed using 2-0 chromic sutures, followed by rectal wall closure with 2-0 Monocryl sutures. Reconstruction of the anal sphincter complex was subsequently performed using 2-0 Monocryl sutures. The operative sequence consisted of fistula tract identification and excision, urethral closure, rectal reconstruction, and sphincter restoration.

**Figure 1:**
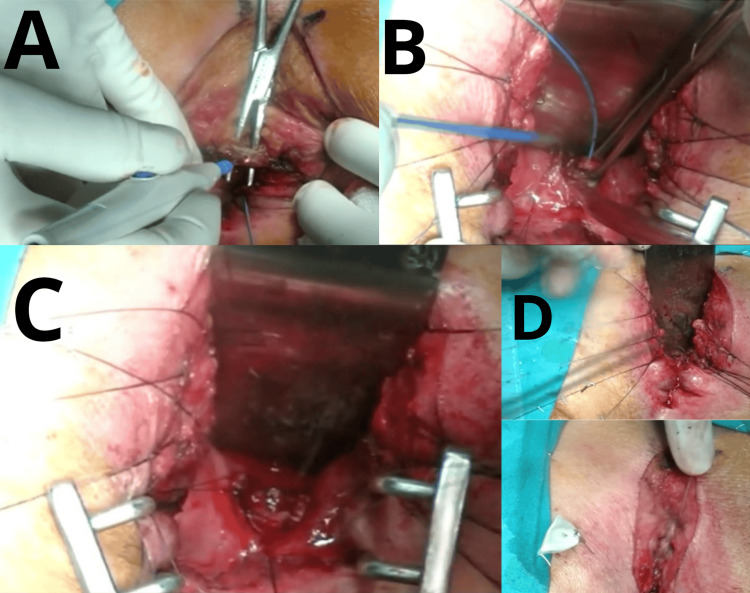
Intraoperative findings during the York-Mason repair. (A) Posterior transsphincteric exposure after coccygectomy. (B) Identification of the rectourethral fistulous tract. (C) Multilayer closure after fistula excision

The rectal wall was then reconstructed, followed by the meticulous reapproximation of the anal sphincter complex using previously placed anatomical landmarks. Skin closure was performed with a continuous absorbable suture. The principal surgical steps of the York-Mason procedure, including exposure, fistula tract cannulation and resection, layered closure of the fistulous tract, and reconstruction of the muscular fascia, are shown in Figure 1.

Due to the patient's extensive previous abdominal surgical history and hostile abdomen, the York-Mason approach was selected as a definitive repair strategy, avoiding additional abdominal dissection and providing direct access to the rectourethral fistulous tract.

Postoperatively, the patient remained hospitalized for three days and was discharged in stable condition. Follow-up contrast enema demonstrated complete rectal integrity without leakage.

At 24 months after the York-Mason repair, the patient maintained normal spontaneous urination and complete fecal continence, with no evidence of fistula recurrence.

Fecal continence was assessed through clinical follow-up evaluation and patient report. The patient denied fecal leakage, urgency, or difficulty controlling bowel movements, and complete fecal continence was clinically documented. A validated continence score, such as the Wexner score, was not applied because this evaluation was performed retrospectively based on available clinical records.

## Discussion

The York-Mason approach, originally described as a posterior transsphincteric route for rectourethral fistula repair, has remained an important surgical option because it provides direct access to the fistulous tract while avoiding extensive abdominal dissection. Its main advantages include excellent visualization, the preservation of surrounding structures, and the possibility of multilayer repair through a controlled surgical field [1]. 

Early surgical descriptions of anorectal and rectourinary fistulous conditions preceded modern reconstructive approaches; however, the management of acquired rectourethral fistulas has evolved substantially with advances in pelvic surgery and reconstructive techniques.

The posterior transsphincteric approach to the rectum was described by Mason in 1970 and was subsequently adopted and modified for the repair of rectourethral fistulas, becoming known as the York-Mason technique [2]. The York-Mason technique is considered by several authors as one of the most effective approaches for rectourethral fistula repair, offering high success rates and low morbidity. It also provides an excellent alternative in patients with a "hostile abdomen" due to multiple prior surgeries, where extensive adhesiolysis may increase the risk of iatrogenic injury [3].

At follow-up, no functional impairment attributable to the additional exposure maneuver was identified. The patient maintained complete fecal continence and satisfactory functional recovery.

Rectourethral fistulas are uncommon but serious complications. They are reported in 1-11% of radical retropubic or perineal prostatectomies and in 0.4-8.8% of cases following brachytherapy, depending on whether it is primary or salvage treatment for prostate cancer [4].

Approximately 60% of rectourethral fistulas are iatrogenic, most commonly resulting from prostatectomy, cryotherapy, radiotherapy, or brachytherapy. Less frequent causes include trauma and inflammatory or infectious diseases. These fistulas significantly increase morbidity and mortality, prolong hospitalization, increase healthcare costs, and markedly reduce quality of life [5,6].

The most common clinical manifestations include dysuria and recurrent cystitis, present in nearly all patients. Pneumaturia and fecaluria are also frequent. Gastrointestinal symptoms such as diarrhea, abdominal pain, and nausea occur in approximately 60% of cases [7].

Although diagnosis can be suspected based on clinical history and physical examination, precise localization of the fistulous tract is often challenging. Imaging and endoscopic studies such as computed tomography (CT) scan, retrograde and voiding cystourethrography, barium enema, cystoscopy, and rectosigmoidoscopy are frequently required. Cystoscopy is considered essential due to its high sensitivity (80-100%).

Treatment depends on etiology, patient condition, surgical expertise, and associated rectal or urinary pathology. There is no universal consensus regarding the optimal management strategy. Surgical options include perineal, transanal, transsphincteric, and combined abdominal-perineal approaches [8].

We present a case in which a transrectal-transsphincteric York-Mason technique was used for the repair of a rectourethral fistula, as an effective alternative in a patient with a hostile abdomen due to multiple prior surgical interventions.

Several surgical techniques exist for the repair of rectourethral fistulas. The choice of approach depends on surgeon experience, fistula location, and previous repair attempts. The management of acquired rectourethral fistulas requires individualized decision-making, considering fistula characteristics, previous treatments, and tissue quality [9].

In patients with multiple prior abdominal surgeries, where adhesiolysis may pose a significant risk of organ injury, alternative surgical approaches to the conventional abdominal route are required.

The York-Mason technique offers the advantages of direct exposure, excellent operative visualization, and low morbidity. Previous reports have demonstrated high success rates with the York-Mason approach, particularly in non-radiated patients and those without extensive tissue destruction [10].

The most critical aspect of this technique is meticulous reconstruction of the anal sphincter complex, which is essential to ensure complete postoperative continence.

In the present case, the choice of the York-Mason approach was influenced by the patient's complex surgical history, including multiple previous abdominal procedures and extensive intra-abdominal adhesions, which made another abdominal approach less favorable. The posterior transsphincteric route allowed direct access to the rectourethral fistulous tract and facilitated multilayer reconstruction with the preservation of anorectal function.

Successful repair of complex rectourethral fistulas depends on several reconstructive principles, including adequate exposure, complete excision of the fistulous tract, tension-free multilayer closure, separation of repaired structures, and preservation of sphincter function. In selected cases, additional maneuvers such as tissue interposition or modifications to improve exposure may be considered according to local anatomy and previous surgical history.

Despite its advantages, the York-Mason approach has important limitations. It requires adequate access to the posterior perineal anatomy and may be less suitable for patients with extensive local fibrosis, severe tissue damage, or fistulas located in unfavorable anatomical positions. Additionally, patients with previous pelvic radiation represent a particularly challenging subgroup because impaired tissue vascularity and healing capacity may increase the risk of repair failure.

Recurrent rectourethral fistulas, especially after previous failed repairs, often require individualized strategies, including tissue interposition, flap reconstruction, or alternative surgical approaches. Abdominal approaches, including transabdominal repair with or without tissue interposition, may be preferred in selected patients with high fistulas, extensive pelvic disease, or associated intra-abdominal pathology. Therefore, the choice of technique should be based on fistula location, etiology, previous interventions, tissue quality, and surgeon experience rather than a single standardized approach.

Reported success rates for rectourethral fistula repair vary according to patient selection, fistula complexity, previous treatment, and surgical technique. The York-Mason approach has demonstrated favorable outcomes in selected patients, particularly those with non-radiated, accessible fistulas. However, comparative studies are limited, and direct comparison between techniques is difficult because published series often include heterogeneous patient populations.

The main contribution of this case is not the description of a novel surgical technique, but rather the demonstration of the applicability of the York-Mason approach in a particularly challenging clinical scenario. This patient presented several factors that increased operative complexity, including multiple previous abdominal procedures, extensive adhesions, and previous failed attempts at fistula management. In this context, the posterior transsphincteric approach provided direct access to the fistulous tract while avoiding additional abdominal dissection.

Additionally, this case emphasizes the importance of individualized surgical planning in complex rectourethral fistulas. The decision-making process should consider fistula etiology, previous interventions, tissue condition, and expected functional outcomes rather than relying solely on a standardized approach.

Vascularized tissue interposition, including gracilis muscle flap, has been described as an important adjunct in selected cases of complex or recurrent rectourethral fistulas, particularly in patients with previous failed repairs, radiation injury, or compromised tissue quality. In the present case, the repair was performed through a York-Mason approach with multilayer closure without tissue interposition. The decision was based on the direct access provided by the posterior transsphincteric approach, the ability to achieve excision of the fistulous tract and separate multilayer reconstruction, and the absence of documented radiation-related tissue damage. The patient's hostile abdomen influenced the choice of a non-abdominal approach but did not specifically determine the use or omission of tissue interposition.

The present case highlights several challenges associated with the management of complex rectourethral fistulas. Previous treatment failure was likely related to the patient's extensive surgical history, including multiple abdominal procedures, previous repair attempts, and the development of a hostile abdomen with significant adhesions. In this context, repeating an abdominal approach would have increased operative complexity and potential morbidity.

The success of the definitive repair in this patient was likely related to several key surgical principles: selection of an alternative non-abdominal approach, direct exposure of the fistulous tract, complete excision of the abnormal communication, and meticulous multilayer reconstruction. The York-Mason approach allowed repair of the urethral defect with 2-0 chromic sutures, rectal closure with 2-0 Monocryl sutures, and restoration of the anal sphincter complex using 2-0 Monocryl sutures.

An important technical consideration in this case was the need for improved operative exposure. Coccygectomy was performed as an adjunctive maneuver due to the complexity of the anatomy and previous interventions. Despite the increased complexity, the patient achieved successful fistula closure with the preservation of fecal continence and the resolution of urinary contamination. This case emphasizes that individualized surgical planning and adaptation of the approach according to previous surgical history and tissue conditions are essential in complex rectourethral fistula repair.

Although robotic-assisted prostate surgery has significantly reduced the incidence of rectourethral fistula compared with historical open approaches, this complication remains clinically relevant because of its significant morbidity and complex management. The present case represents an uncommon scenario in which a fistula developed after robotic-assisted prostate surgery for benign prostatic hyperplasia, followed by a complex postoperative course requiring multiple interventions. The combination of previous surgical attempts, intra-abdominal complications, and extensive adhesions contributed to the complexity of subsequent management.

## Conclusions

The York-Mason technique remains a valuable surgical option for selected patients with rectourethral fistula, especially when previous abdominal surgeries increase the complexity and risk of conventional approaches. Rectourethral fistula repair remains a complex surgical challenge requiring individualized treatment strategies. In patients with hostile abdomens due to multiple previous surgeries, the York-Mason transsphincteric approach represents a valuable alternative that provides direct exposure, avoids extensive adhesiolysis, and allows successful repair with preservation of continence. Although larger studies are required, this technique continues to be an important option in selected patients when conventional abdominal approaches involve increased risk.
